# PD135 Real-World Evidence On The Effects Of Robotic Prostatectomy In Poland

**DOI:** 10.1017/S0266462324003726

**Published:** 2025-01-07

**Authors:** Maciej Dzik, Aneta Płusa, Monika Zaleska

## Abstract

**Introduction:**

Robot-assisted radical prostatectomy (RARP) was incorporated into the public healthcare system in Poland in April 2022. RARP quickly gained popularity among healthcare providers, constituting nearly 25 percent of all publicly financed prostatectomies by the end of 2022. The aim of this study was to evaluate the effects of RARP using early real-world data from the public reporting system.

**Methods:**

The sample included 7,177 patients who had either RARP or conventional radical prostatectomy (CRP) between 27 March and 31 December 2022. CRP was performed as either an open or a laparoscopic procedure. Due to reporting limitations, a comparison with laparoscopic radical prostatectomy (LRP) only was carried out on a subset of 2,306 patients who had prostatectomy after 20 September 2022. Data analyzed included length of hospitalization, the percentage of patients who received transfusions of blood products or who were hospitalized within 30 days of discharge, and number of deaths.

**Results:**

In total 2,190 patients had RARP. Compared with both CRP and LRP, RARP was associated with a reduction in hospital stay by 1.13 days (95% confidence interval [CI]: −1.27, −0.99; p<0.0001) and 0.83 days (95% CI: −1.02, −0.64; p<0.0001), respectively, and a lower risk of needing a transfusion of blood products, with odds ratios of 0.39 (95% CI: 0.31, 0.49; p<0.0001) and 0.53 (95% CI: 0.39, 0.77; p=0.0008), respectively. There were no statistically significant differences in rates of rehospitalization. Only three hospitalizations ended due to death. By 31 December 2022 only seven patients in the RARP group and 19 in the CRP group had died.

**Conclusions:**

The findings of this study suggest that there is a marginal, though statistically significant, benefit with RARP, compared with CRP and LRP, that may be factored into economic evaluations of RARP.

